# A Case Report of Baclofen- and Clozapine-Induced Dyskinesia: A Movement Disorder

**DOI:** 10.7759/cureus.25068

**Published:** 2022-05-17

**Authors:** Maisha Maliha, Zinath Roksana, Priyata Dutta, Md Y Mamoon, Mohammed Q Islam

**Affiliations:** 1 Internal Medicine, Dhaka Medical College, Dhaka, BGD; 2 Internal Medicine, Feni Sadar Hospital, Feni, BGD; 3 Internal Medicine, University of Michigan, Ann Arbor, USA; 4 Internal Medicine, Queens Hospital Center, New York City, USA; 5 Neurology, Queens Hospital Center, New York City, USA

**Keywords:** drug interaction, movement disorder, dyskinesia, clozapine, baclofen

## Abstract

Movement disorder is a broad term comprising multiple disorders which result in either an excess or a paucity of voluntary and involuntary movements. There are numerous pieces of literature on drug-induced dyskinesia, although the exact mechanism underlying this phenomenon is yet to be understood. Drug-induced movement disorder is a complex and often neglected clinical presentation. There are various interactions of drugs with the dopaminergic, GABAergic, and serotonergic pathways in the body that seem to be the foundation, leading to these movement disorders. Further research and clinical trials are required to understand this clinical entity. Here we present a case report of GABAergic baclofen and an anti-dopaminergic clozapine-induced atypical case of dyskinesia, a severe form of movement disorder in a 69-year-old-male with a past medical history of physiologic tremor and neuropathic pain.

## Introduction

The term “movement disorder” denotes abnormality in movement, which may be either voluntary or involuntary [1]. There are various movement disorders: dystonia, tremor, myoclonus, tardive dyskinesia, hemiballismus, tics, chorea, athetosis, and others [2]. Multiple etiologies of movement disorders include genetics, toxins, metabolic abnormalities, infections, strokes, and vascular complications [1]. Among them, drug-induced dyskinesia is one of the leading causes of movement disorder [3]. In a constantly changing population using widespread polypharmacy, identifying the combination of drugs that can cause tremors is very important to generate risk profiles for individual patients [3]. The drugs implicated in movement disorder are antipsychotics, CNS stimulants, antidepressants, anticonvulsants, antiparkinsonian drugs, bronchodilators, amiodarone, and lithium [4]. However, a combination of skeletal muscle relaxants and second-generation antipsychotics such as baclofen and clozapine-induced movement disorder, especially dyskinesia, is a rare incident [5]. Risk factors for drug-induced movement disorder include polypharmacy, male sex, older age, an increased dose of medicine, and extended-release drug formulations that quickly reach high toxic levels in the body [6]. Herein, we present a case report of a 69-year-old male who presented with clozapine and baclofen-induced dyskinesia, a rare adverse effect of these medications.

## Case presentation

A 69-year-old Asian American man with a past medical history of physiologic tremor, hyperlipidemia, and hypertension, presented to our clinic for his regular checkup. He recently migrated to the United States of America six months ago. For 30 years, he has had a history of involuntary rhythmic jerking movement of his entire body, most significantly in the neck, back, and hands. There are no exacerbating factors, but it is relieved by sleeping. However, the movement persists in the same intensity and frequency throughout the day and causes significant functional impairment in his daily activities. Initially, he was prescribed diazepam, which resulted in no significant improvement. Consequently, he was prescribed by his primary care physician back in his home country to take 25 mg of clozapine and 10 mg of baclofen for physiologic tremor and neuropathic pain, respectively. The patient has been taking these two drugs for the last 30 years. Other medications include lisinopril and atorvastatin.

The patient does not have a family history of neurological or movement disorders, including Sydenham chorea, Huntington’s disease, and Wilson’s disease. In addition, he had no significant triggers for developing physiologic tremors. The patient was alert and oriented with intact short- and long-term memory on examination. His vitals were within normal limits. He exhibited rhythmic involuntary jerking movement, most prominently in the neck, back and hands, as seen in Video 1.

**Video 1:** Dyskinesia caused by baclofen and clozapine.

Additional findings from the nervous system examination were unremarkable. Other systematic examination findings were normal. The patient’s brain MRI revealed no significant findings. Investigations such as electroencephalogram, electromyography, and transcranial Doppler ultrasound were normal. Likewise, his routine laboratory findings were within normal limits. We stopped baclofen and clozapine and prescribed gabapentin 100 mg with an appropriate follow-up visit scheduled in four weeks.

## Discussion

Our patient has been taking baclofen and clozapine for his neuropathic pain and physiologic tremor, respectively, for the last 30 years. Baclofen, a muscle relaxant, acts as a GABA receptor agonist, decreasing excitatory neurotransmitter release from presynaptic terminals [7]. It is the drug of choice in treating multiple sclerosis, spinal cord injuries, and other musculoskeletal disorders [8]. The most reported adverse effects of baclofen use include sedation, lethargy, vertigo, seizures, and hyperthermia [9]. Other less reported side effects include psychic symptoms and muscular dyskinesia [5].

Clozapine exerts its effects by blocking 5-HT2A serotonin and D2 dopamine receptors, with some blockade of norepinephrine alpha-2 receptors [10]. It is primarily used to treat multidrug-resistant schizophrenia [11]. Clozapine is also prescribed to treat delusional disorders, bipolar disorders, and mania [10]. Adverse effects of this drug include extrapyramidal effects such as akinesia and dystonia, hematological disorders, and seizures [12]. Although our patient was taking a lower dose of clozapine for his physiologic tremor, lower clearance or higher drug concentration to dose ratio associated with Asian ethnicity, obesity, poor genetic metabolizer with renal impairment could be one of the reasons for the observed side effects [13].

**Figure 1:**
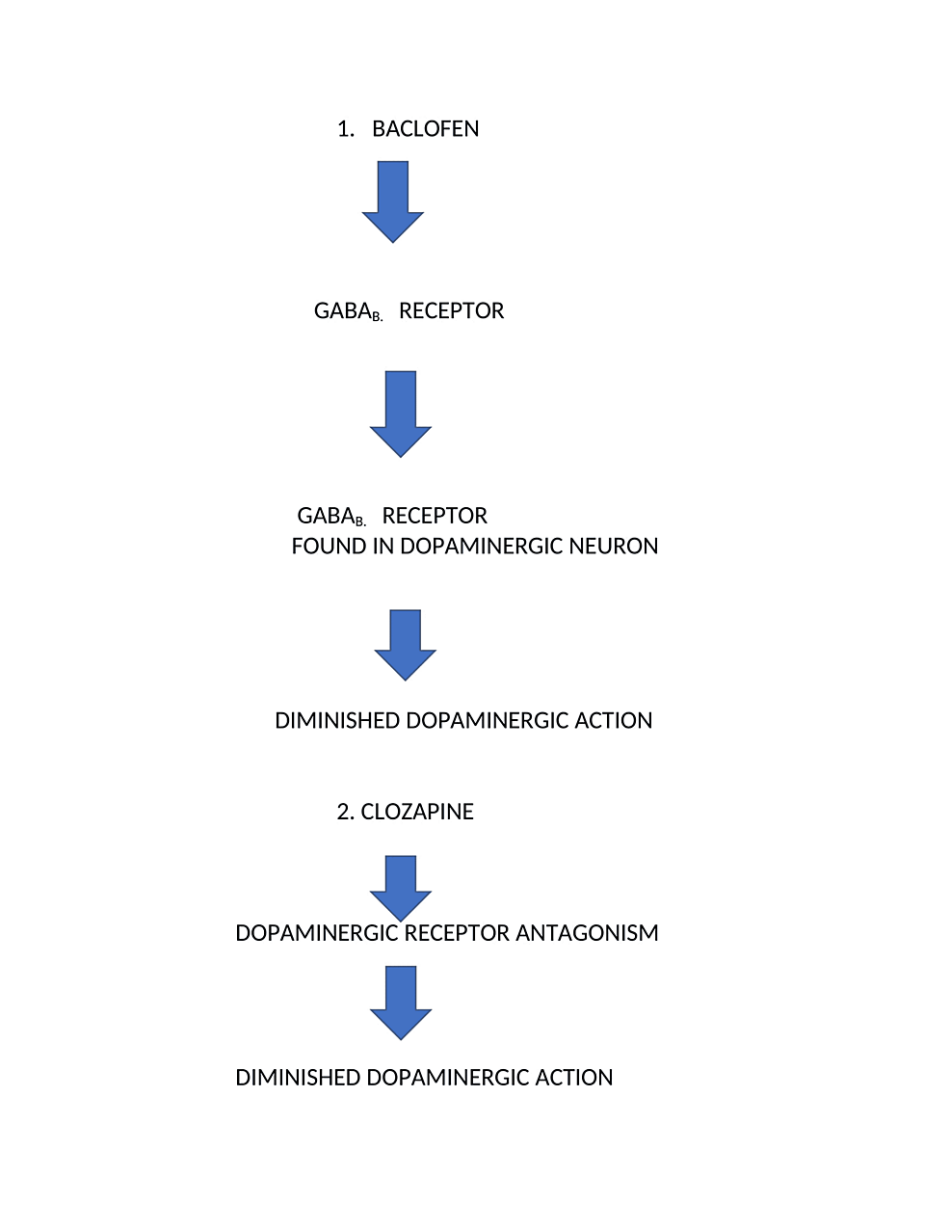
Baclofen and clozapine action on dopamine.

The exact mechanism of baclofen and clozapine-induced dyskinesia is poorly understood. It has been hypothesized that relative dopaminergic hypofunction caused by second-generation antipsychotic clozapine may be augmented by baclofen [7]. Some rodent models have shown that GABA receptors are expressed on both dopaminergic and GABAergic neurons [7]. Therefore, exposure to baclofen may cause activation of GABA receptors and inhibit the release of both GABAergic and dopaminergic neuron activity, modifying the release of both GABA and dopamine [7]. So hence we theorize that in our case, a combination of baclofen and clozapine resulted in enhanced, diminished dopaminergic activity. This led to the dysfunction of the nigrostriatal pathway and the development of this debilitating movement disorder in our patient, as shown in Figure 1.

Although very few articles have discussed the combination of baclofen and clozapine use, simultaneous use of these drugs can positively accentuate these adverse side effects. Therefore, this report can raise awareness among physicians and guide them on proper drug choice and prescription.

## Conclusions

Physicians should be aware that using a combination of antipsychotic and muscle relaxants such as clozapine and baclofen for a prolonged period for neuropathic pain and physiologic tremor can lead to the development of dyskinesia. Our case report provides an excellent example of how using such a combination can result in a movement disorder that significantly impairs quality of life. The combined effect of these drugs on dopaminergic and GABAergic neurons can profoundly impact the nigrostriatal pathway. Given the significant movement disorder that developed in this case, we strongly suggest exploring more about such drug-drug interaction cases that can guide the physicians and make them mindful of this interaction while prescribing drugs for the patients in the future.
